# Stakeholder Perceptions on Innovative Private Pharmacy Distribution Channels and Implications for Medicine Quality in Zambia: A Qualitative Study

**DOI:** 10.9745/GHSP-D-24-00248

**Published:** 2026-08-10

**Authors:** Scott Kaba Matafwali, Virginia Bond, Sian E. Clarke, Harparkash Kaur

**Affiliations:** a Department of Clinical Research, London School of Hygiene and Tropical Medicine, London, UK.; b Department of Global Health and Development, Faculty of Public Health and Policy, London School of Hygiene and Tropical Medicine, London, UK.; c Zambart, School of Public Health, University of Zambia, Ridgeway Campus, Lusaka, Zambia.; d Department of Disease Control, London School of Hygiene and Tropical Medicine, London, UK.

## Abstract

**Introduction:**

Innovative private pharmacy distribution channels, including delivery services, telepharmacy, and e-pharmacies, have emerged as potential solutions to improve access to quality-assured medicine and pharmacy services in Zambia, but limited evidence exists regarding their impact on medicine quality and safety. This qualitative study provides the first comprehensive investigation of stakeholder perspectives on these channels, examining their implications for pharmaceutical quality assurance and distribution in Zambia’s health care system.

**Methods:**

A purposive sample of 15 stakeholders participated in the study, selected to represent key sectors of the pharmaceutical supply chain, including wholesalers, innovators, retailers, national medicine supply agencies, national medicine regulators, professional pharmacy bodies, and supply chain experts. Semi-structured interviews were conducted until data saturation was achieved. Interviews were recorded and transcribed, and a bottom-up thematic analysis was employed to derive themes directly from the data.

**Results:**

Themes that emerged from the analysis fell into 4 interconnected categories of issues: (1) current challenges in the pharmaceutical supply chain (including affordability barriers, transportation limitations, and regulatory constraints) affecting both traditional and innovative pharmacy services, (2) potential benefits of the innovative pharmacy approaches (such as enhanced traceability and improved quality assurance), (3) limitations and shortcomings (including technical and behavioral challenges), and (4) recommendations for improvement. Stakeholder perspectives varied notably by role, with regulators emphasizing quality control and innovators focusing on accessibility and efficiency.

**Conclusion:**

The findings suggest that innovative pharmacy distribution approaches will be constrained by some of the same regulatory and structural issues faced by traditional pharmacy systems, and thus cannot fully circumvent existing challenges of access to quality pharmacy services and medicines in Zambia and other countries. To successfully realize the potential of pharmacy innovations to improve accessibility to good-quality medicines, balanced attention is required to technological advancement, regulatory compliance, and the local context. These insights offer actionable guidance for policymakers and stakeholders seeking to strengthen medicine distribution systems, enriching the broader narrative of health care delivery in resource-limited settings.

## Abstract (precis)

Innovative pharmacy channels in Zambia offer benefits, such as enhanced traceability and improved quality assurance, but also face challenges, including slow adoption of technology, financial barriers to innovation, and regulatory hurdles. A 3-pronged approach involving technological integration, government support, and robust regulatory frameworks is recommended to realize the full benefits of such innovative distribution channels and ensure access to quality medicines.

## Teaser

**Key Findings:**

- Innovative private pharmacy distribution channels in Zambia and other countries have the potential to improve medicine access, affordability, and quality but also face unique challenges. In-depth interviews were conducted with stakeholders in Zambia to explore their perceptions of the potential impact of these channels.
- Innovative pharmacy distribution channels in Zambia were seen to offer potential benefits such as increased product availability, improved quality assurance, and enhanced traceability of medicines.
- Stakeholders also identified challenges related to affordability, transportation, availability, substandard medicines, and regulatory issues, similar to those previously reported to constrain traditional approaches.
- Behavioral, financial, regulatory, and technical considerations must be addressed to fully realize the potential of innovative approaches.

**Key Implications:**

- For policymakers: A supportive regulatory framework and government involvement are needed to foster the effective implementation of innovative pharmacy channels.
- For program managers: Education and training for government and pharmaceutical stakeholders are important to ensure the successful adoption and utilization of innovative technologies and practices.
- For researchers: Further research on the impact of innovative pharmacy channels on medicine quality and patient outcomes in Zambia and other low- and middle-income countries is warranted.

## INTRODUCTION

Innovative private pharmacy distribution channels represent novel and alternative methods of delivering medicines to enhance accessibility, affordability, and quality.^1^^–^^3^ These channels leverage technology to optimize the supply chain and increase efficiency and transparency. Examples include mHealth solutions for direct medicine delivery, e-commerce platforms connecting pharmacies and manufacturers to consumers, and last-mile delivery services using drones.^1^^,^^4^^–^^6^ Innovations extend beyond delivery technology to include inventory management systems, franchise partnerships, and medicine authentication systems at the pharmacy retail level, aiming to reduce costs and improve availability.^2^ Other innovative approaches include social enterprise pharmacies that reinvest profits to enhance health care infrastructure and services in underserved communities and community-based pharmacies employing local health care workers to provide patient education and counseling.^7^^,^^8^ These initiatives operate in various countries, including Ghana, Kenya, Nigeria, and Zambia.^1^^,^^2^^,^^9^

The pharmaceutical sector in Zambia faces significant structural and operational challenges that fundamentally impact medicine access and quality. The country’s pharmaceutical market operates with severely limited domestic manufacturing capacity, producing less than 10% of domestic demand.^10^^,^^11^ This limitation has led to substantial reliance on pharmaceutical imports, with over 90% of essential medicines sourced from international markets. Distribution networks have evolved into complex systems with multiple intermediaries, creating additional challenges in medicine delivery and quality assurance. Rural areas face particularly acute challenges in last-mile delivery while resource constraints throughout the system hamper effective regulatory oversight and quality control.^10^^,^^12^^,^^13^ These intersecting challenges have created an environment where innovative solutions are increasingly necessary to ensure reliable access to quality-assured medicines.

In the Zambian context, several companies have emerged as pioneers in innovative pharmaceutical distribution. Organizations including mPharma, VIA Global Health, Right ePharmacy, and HnG Online Pharmacy have implemented various technological solutions to address distribution challenges, such as bespoke inventory management software, vendor-managed inventory services, direct-to-consumer distribution systems, telemedicine platforms, and product authentication services.^1^^,^^9^ For example, mPharma manages multiple pharmaceutical inventories in Lusaka through a franchise mechanism, while Right ePharmacy has established parcel collection units and positioned itself as a potential provider for centralized medicine distribution systems.^1^^,^^12^

The regulation of innovative pharmacy channels in Zambia operates within the framework established by the Medicines and Allied Substances Act No. 3 of 2013.^14^ While traditional pharmacy operations are well-defined within this regulatory structure, innovative channels currently operate in a partially regulated space. The Zambia Medicines Regulatory Authority (ZAMRA) has begun developing guidelines for e-pharmacy and telepharmacy services, though comprehensive regulations continue to evolve.^12^ Current quality assurance requirements mandate registration of all pharmacy operations and establish standards for proper storage and handling of medicines. These requirements include detailed standard operating procedures for temperature-sensitive products and comprehensive documentation for product traceability. However, significant regulatory gaps persist, particularly regarding digital health services and remote dispensing. Guidelines for electronic prescription handling remain unclear, and frameworks for digital authentication systems are still under development.^1^^,^^9^^,^^12^

While innovative pharmacy approaches aim to address issues in the conventional supply chain, they also face their own unique set of challenges. Understanding both the potential benefits and limitations of these approaches is crucial for developing comprehensive solutions to improve medicine access and quality in Zambia. Concerns have been raised regarding the quality and safety of medicines sold through these different channels, particularly where proper regulatory oversight may be lacking.^1^^,^^2^^,^^15^ The absence of specific regulatory frameworks for innovative distribution channels in low- and middle-income countries (LMICs) could make pharmaceutical supply chains vulnerable to substandard and falsified medicines, compromising patient safety.^15^

Through in-depth interviews with a range of key stakeholders, this qualitative study aimed to explore perceptions of innovative private pharmacy distribution channels currently operational in Zambia. Specifically, this study sought to identify the perceived advantages and disadvantages of these channels in relation to the quality and safety of the medicines sold through them. By understanding stakeholder perspectives, this study aimed to provide valuable insights into how these innovative pharmacy distribution channels can be regulated and monitored to ensure public access to quality medicines.

## METHODS

### Study Design

This qualitative study was undertaken as part of a PhD research project that aimed to compare the quality of medicines (antimalarials and antibiotics) available in innovative pharmacies with those available in independent pharmacies using traditional supply chains.^12^ The qualitative research component employed semi-structured interviews to allow participants to express their views and experiences in their own words while ensuring coverage of key topics related to medicine quality and distribution.^16^^–^^18^ This method is suitable for collecting information and contextual details regarding complex problems in qualitative research. The qualitative methodology enabled detailed exploration of stakeholder experiences and perspectives, providing crucial context for understanding how innovative distribution channels operate within Zambia’s pharmaceutical landscape.

### Study Setting

The study was conducted in Zambia in October to November 2022, with key informant interviews conducted primarily in Lusaka, the capital city, and one international location, to capture a diverse range of perspectives and experiences relating to innovative private pharmacy distribution channels and the quality of medicines in Zambia. Lusaka was selected as the primary study location due to its role as the country’s economic and administrative center, home to approximately 3 million residents.^19^ Lusaka presents a unique context for studying pharmaceutical innovation, given its relatively higher average household income, education levels, and health care service access compared with other provinces in Zambia.^20^ The city has a mix of public and private health care facilities including hospitals, clinics, and pharmacies. Independent pharmacies, which are privately owned and not affiliated with a hospital or clinic, are a significant source of medicine for many residents of Lusaka.^21^ This diverse health care landscape provided an ideal setting for examining the implementation and impact of innovative private pharmacy distribution channels. At the time of the study, all such pharmacies in Zambia were concentrated in Lusaka, with the first innovative pharmacy chain established in 2013.

### Innovative Private Pharmacy Distribution Channels in Zambia

For this study, we defined the 'Innovative Pharmacy Approach' as the use of technology-enabled solutions to improve pharmaceutical distribution (Table 1). These solutions encompass a range of services and technologies, including technology-driven services for hospitals, clinics, and pharmacies, such as pharmacy inventory-management software, vendor-managed inventory services, and business-to-business (B2B) marketplace platforms. Additionally, they cover tech-enabled services for patients, including direct-to-consumer distribution, telemedicine, product locators, and patient engagement tools. The approach also incorporates product data and medicines authentication services, encompassing falsified medicine screening, track-and-trace systems, and data analytic services. Participants were provided with this definition and examples before discussing their perceptions and experiences.

**TABLE 1.** Examples of Innovative Pharmacy Approaches Grouped by Beneficiaries

**Beneficiary**	**Examples of Innovative Pharmacy Approaches**
Regulators	Track-and-trace systems Falsified medicine screening Data analytics services
Providers	Pharmacy inventory-management software Vendor-managed inventory services Business-to-business marketplace platforms
Patients	Direct-to-consumer distribution Telemedicine Product locators Patient engagement tools

### Sampling and Participants

The study employed purposive sampling to ensure qualitative representation of key stakeholder groups in Zambia’s pharmaceutical sector. The final sample comprised 15 participants representing multiple sectors: wholesalers (n=3), innovators (n=5), retailers (n=3), national medicine supply agencies (n=1), national medicine regulators (n=1), professional pharmacy bodies (n=1), and supply chain experts (n=1). Selection criteria for participants included direct involvement in or oversight of pharmaceutical distribution and at least 5 years of experience in their current role for most participants, with knowledge of or experience with innovative distribution channels. Data saturation was achieved after 13 interviews, with 2 additional interviews conducted to confirm no new themes emerged. Initial participants were identified through professional networks and recommendations from initial interviewees (snowball sampling). They were contacted and invited to participate. One participant representing an innovative organization declined due to time constraints, but this did not impact the achievement of data saturation.

### Data Collection

The in-depth interviews were conducted in English and ranged in duration from 20 to 60 minutes. The interviews were typically conducted at the interviewee’s workplace in a private office, with only the participant and researcher present. Due to logistical constraints, 3 interviews were conducted over Zoom and 3 others at a nearby quiet cafeteria. Written informed consent was obtained from each participant prior to the interviews. The interviews were conducted by SKM, the first author.

Two distinct interview guides were designed to address the different roles and experiences of participants. The first guide was tailored for wholesalers, retailers, and innovators who had direct contact with the medicines while the second guide addressed perspectives of regulatory and professional bodies, whose interactions with medicines are indirect, thereby necessitating a different set of questions.

The interviews were recorded and transcribed with the participants’ consent. However, in 3 cases, notes were taken instead of recordings because the participants were uncomfortable with the recording. After each interview, the transcriptions were reviewed by SKM to ensure accuracy. The final transcripts were then cross-checked against the original notes and recordings.

### Data Analysis

Interview data were managed using NVivo version 12 (QSR International Pty Ltd.). Transcripts were initially coded independently by SKM and a fellow social science PhD student with substantive prior experience in qualitative research. Following this independent coding process, SKM then reviewed and made final adjustments to the codes to ensure consistency and accuracy.

The qualitative data collected from the in-depth interviews were analyzed using a thematic analysis approach, as outlined by Braun and Clarke.^22^^,^^23^ Thematic analysis is a flexible and widely used approach for analyzing qualitative data that allows the identification of patterns and themes that emerge from the data itself. This is particularly useful in exploring complex phenomena.

The analysis began with familiarization with the data by reading and re-reading the transcripts. The initial codes were then generated from the data and grouped into themes based on the identified patterns. A constant comparison was used to ensure that the themes accurately represented the data and that they were distinct from one another. The themes were then refined, reviewed, and defined to produce a clear narrative relevant to the research questions. As the analysis progressed, the researchers engaged in reflective discussions to ensure that the themes were grounded in the data and aligned with research objectives. To provide an external check on the data and analysis process, the themes and subthemes were critiqued and validated by an experienced qualitative researcher (VB).

### Ethical Considerations and Reflexivity

This study was conducted with ethical approval from the Biomedical Research Ethics Committee (REF. No 2926-2022) and the National Health Research Authority (NHRA000010/10/07/2022) in Zambia, as well as London School of Hygiene and Tropical Medicine Ethics Committee (Ref:28040) in the UK. Written informed consent was obtained from all participants who participated in this study. Participant anonymity was protected through consistent reassurance about confidentiality, especially when participants expressed concerns about identity disclosure. The management of power dynamics was given specific consideration throughout the data collection process by, for example, using lay language instead of technical pharmaceutical terminology, allowing participants to choose interview locations, and emphasizing their expertise in their respective roles.

Particular attention was also given to the unique position of the first author’s (SKM) role as a Zambian pharmacist, which may have influenced the interview dynamics, if participants viewed the researcher as a knowledgeable insider, potentially affecting their responses. This professional lens could also introduce biases into the study, influencing both the questions posed and interpretation of the data. To address these considerations, specific reflexivity measures were implemented. For example, during interviews, the researcher sought to maintain a neutral stance by using open-ended questions and avoiding technical pharmaceutical terminology that might influence responses. For instance, when discussing medicine quality, rather than using professional pharmaceutical terms, participants were asked to describe their experiences in their own words. Interview transcripts were reviewed with non-pharmacist researchers who highlighted instances where professional knowledge might have influenced the interpretation, enabling reanalysis from a more neutral perspective. Additionally, when analyzing responses about regulatory challenges, the researcher initially interpreted certain stakeholder criticisms through a pharmacist’s lens of professional standards. However, through reflexive journaling and team discussions, these interpretations were reexamined to ensure they reflected participants’ actual views rather than professional assumptions. In another instance, when participants discussed quality assurance practices, the researcher documented potential preconceptions about proper pharmaceutical procedures before analyzing the data, allowing for more objective interpretation of participants’ actual experiences.

## RESULTS

Analysis of interviews with 15 stakeholders revealed patterns of perspectives that both aligned and diverged across different professional roles. As shown in Table 2, the participant group comprised wholesalers (n=3), innovators (n=5), retailers (n=3), regulatory bodies (n=3), and supply chain (n=1), including a key informant from each of the following: national medicine supply agency, national medicine regulator, professional pharmacy body, and supply chain experts. Most participants had 5–9 years of experience or more than 14 years in their respective fields, with over half holding at least a master’s degree.

**TABLE 2.** Characteristics of Stakeholders Interviewed, Lusaka, Zambia, 2022

**Characteristic**	**No. of Participants**
Role	
Leadership	8
Operational	3
Managerial	4
Sector	
Wholesale	3
Innovator	5
Retailer	3
Regulator	3
Supply chain	1
Education level	
Bachelors	6
Masters	8
PhD	1
Years of experience	
5–9	6
10–14	1
>14	8

Four main categories emerged from the thematic analysis (Table 3):

**TABLE 3.** Summary of Themes and Subthemes From Stakeholder Interviews, by Main Category, Lusaka, Zambia, 2022

**Category**	**Themes**	**Subthemes**
Challenges in the pharmaceutical supply chain	Affordability of medicines	Medicines are expensive
		Stockouts of medicines
		Monopolies are barriers to access
	Poor transportation	
	Presence of poor-quality medicines	
	Regulatory challenges	Practice and regulation mismatch
		Regulation impacts access and affordability
		Rigid licensing guidelines
		Availability of medicines
		Slow regulatory process
Potential benefits of the innovative pharmacy approach	Improved availability of products	Fast track product registration
		Improved access and distribution
		Improved economy
		Improved procurement efficiency
		Improve supply chain bottlenecks
	Improved quality assurance	Authentication of products
		Improved monitoring of products
	Improved traceability	Improved track and trace
Limitations and shortcomings to the innovative pharmacy approach	Behavioral challenges	Lack of awareness
		People are slow to change
		Reluctant acceptance of technology
		Resistance to change
	Financial challenges	High cost of innovation
		Lack of financial support
	Lack of government interest	Lack of involvement by the Ministry of Health
		No government support
	Quality assurance challenges	Lack of authentication technologies
	Regulatory challenges	Lack of specific regulations
		Rigid pharmaceutical regulations
	Technical challenges	Electricity and internet as barriers
		Innovation is not inclusive
		Last point delivery confusion
		Lead time challenges
Recommendations for improvement	Digitization	Digitize patient records
		Increased digitization of the country
	Education	Increased training for regulators
		Increased awareness
	Government lead	Introduce more technology
		Investment into technology
	Quality assurance	Leverage technology
		Need innovation for authentication
	Regulation	Need for a new regulatory framework
		Need for localized regulations
		Need for regulation to enforce medicine quality
	Research

- Current challenges in the pharmaceutical supply chain (which affect both traditional and innovative pharmacy distribution channels)
- Potential benefits of the innovative pharmacy approach
- Limitations and shortcomings to the innovative pharmacy approach
- Recommendations for improvement

The majority of participants—including all innovators, wholesalers, and retailers—provided insights based on direct operational experiences with innovative approaches. The remaining participants (n=4), primarily from regulatory and professional bodies, shared perspectives from an oversight position rather than direct use or implementation. This stakeholder distribution ensured a balanced representation of both hands-on implementation experience and regulatory oversight perspectives.

### Challenges in the Pharmaceutical Supply Chain

#### Affordability of Medicines

The majority of participants underscored several key challenges that affect both traditional and innovative pharmacy channels, such as the affordability of medicines, with several participants noting that “medicines are just too expensive.” Frequent stockouts of medicines were also highlighted, which indirectly relates to affordability because when medicines are out of stock, patients may be compelled to buy the same drugs at higher prices elsewhere or resort to less effective but more affordable alternatives.

*Stockouts of medicines are a common occurrence, forcing patients to either delay treatment or pay higher prices at alternative outlets. —Participant 4, retailer*

#### Regulatory Challenges

Nine participants, including all wholesalers and most innovators, identified regulatory challenges that impacted the pharmaceutical supply chain. Participants identified regulations that inadvertently favor monopolies, creating a barrier to access:

*The regulation requires that one particular pharmaceutical company brings a particular brand of a drug, limiting competition and accessibility. This creates a monopolistic situation where a single company controls the supply of a specific medication. As a result, prices can be high, and patients may struggle to access the medicines they need. If there were more competition in the market, with multiple companies offering the same drug, it could lead to lower prices and improved availability. However, the current regulatory framework seems to favor these monopolies, which is a significant barrier to ensuring affordable and accessible medicines for all. —Participant 14, wholesaler*

Other participants mentioned a “mismatch between practice and regulation,” where regulations were not aligned with the needs of patients and health care providers. For instance, one participant noted the requirement for a pharmacist to be present in each physical pharmacy location, which could pose a challenge in areas with pharmacist shortages and limit the accessibility of medicines in these regions. Strict licensing guidelines and slow regulatory processes were also cited as additional challenges impacting the access to and affordability of medicines.

#### Poor Transportation Infrastructure

The inadequate transportation infrastructure, especially in rural regions, poses challenges to medicine delivery and quality maintenance. Several participants highlighted infrastructure challenges, with two specifically mentioning the need for broader consideration:

*We also need to think about how to cater to people outside Lusaka, especially with the bad roads … and at the same time, maintaining the quality of those medicines. Because they are not common goods. —Participant 5, retailer*

#### Poor-Quality Medicines

Seven participants identified the suspected presence of poor-quality medicines as a concern. Several participants mentioned that falsified products are a pressing issue, and they are unsure of what they give patients.

*Counterfeit products, especially with the distribution process that is not very well regulated, is resulting in a lot of counterfeit products around. When there are gaps in the regulation and oversight of the distribution chain, it creates opportunities for counterfeit medicines to enter the market. These fake products can be difficult to distinguish from genuine ones, and they pose a serious risk to patient safety. … Strengthening the regulation and monitoring of the distribution process is crucial to combat the proliferation of counterfeit medicines and protect public health. —Participant 10, retailer*

Another participant pointed out that:

*our economy is always about the cheapest, but the problem is that in being cheap, most of the time, what we are finding [is] counterfeit medication. —Participant 13, innovator*

### Potential Benefits of Innovative Pharmacy Approaches

#### Availability and Distribution

Ten participants highlighted some potential benefits of the innovative pharmacy approach, with the strongest support coming from innovators and retailers, noting that innovative approaches, particularly those that reduce intermediaries in the supply chain or deliver medicines directly to patients, could enhance product affordability and availability.

A few participants also underscored the role that the innovative pharmacy approach could play in overcoming numerous supply chain bottlenecks, such as the presence of many intermediaries and challenges in medicine transportation. As one participant expressed, these approaches could improve accessibility:

*[the innovative approaches] improve accessibility to medical products through improving the distribution process … —Participant 2, innovator*

Furthermore, the notion of fast-tracking product registration using innovative technologies was raised by 7 participants. This was viewed as a potential means to increase access and distribution, bolster the economy, and enhance procurement efficiency. One participant explained that by simplifying and accelerating the registration process, multiple manufacturers could be encouraged to enter the market, thereby expanding the range and availability of medicines:

*Fast-tracked product registration could stimulate multiple manufacturers to register products within a country, thereby increasing the variety and availability of medicines. —Participant 5, retailer*

#### Quality Assurance and Traceability

Enhanced quality assurance, particularly product authentication and monitoring, emerged as another potential benefit. It was pointed out that the introduction of technology could be instrumental in improving the quality of medicines in several ways.

*The innovative pharmacy approach can significantly enhance quality assurance by leveraging technology for product authentication and monitoring. By introducing checks at multiple points along the supply chain, we can verify the genuineness of medicines and ensure that only high-quality products reach the patients. This technological integration is a game-changer in our efforts to combat counterfeit drugs and maintain the integrity of the pharmaceutical supply. —Participant 6, regulator*

Improved traceability was also identified as a key benefit by 8 participants, with participants envisaging a system in which an application could enable the real-time tracking of medicine delivery from the pickup point to the final delivery destination. This could facilitate a seamless ordering and delivery process for retail pharmacies, leading to an efficient, real-time delivery of orders.

*One of the key advantages of the innovative pharmacy approach is the potential for improved traceability. … Imagine a system where we can track medicines in real-time, right from the point of pickup to the final delivery destination. This level of visibility would be revolutionary, enabling us to monitor the movement of medicines at every step. For retail pharmacies, this could streamline the ordering and delivery process, ensuring a more efficient and transparent supply chain. —Participant 11, regulator*

### Limitations and Shortcomings to the Innovative Pharmacy approach

#### Behavioral Challenges

Eight participants identified behavioral challenges, with lack of awareness and slow adoption of technology among regulators, government, and some sectors of the pharmaceutical business being key issues. While some participants did not explicitly discuss resistance to change, the slow adoption of technology can be seen as an implicit form of resistance due to reluctance or unfamiliarity with new systems. However, some participants expressed positive views about embracing change in the sector:

*Innovation is never kept, because you cannot put a cap on ideas and the mind. It’s always encouraged that people should think through better ways of doing business. —Participant 8, wholesaler*

#### Financial Barriers

Ten participants cited financial challenges as significant barriers, particularly the high cost of innovation and the lack of financial support from the government. One participant commented on these barriers, saying:

*The major challenge is just no support whatsoever, lack of capital, lack of financial support, lack of structural support. —Participant 2, innovator*

The lack of government interest and involvement in the innovative pharmacy approach was a concern for participants. As one participant stated:

*It's a lonely world out there, because there’s basically no support from government and no supportive environment. —Participant 2, innovator*

#### Regulatory Hurdles

Regulatory challenges emerged as a key theme, with participants identifying issues at both ends of the spectrum. Some participants expressed concerns regarding the lack of specific regulations, particularly in emerging areas such as online and telepharmacy services. However, the existing pharmaceutical regulations are perceived as too rigid, potentially hindering innovative practices.

*We need robust systems to help regulators enforce the law, especially regarding online and telepharmacy services. … Without clear guidelines and tools to monitor these services, there is a risk of substandard or counterfeit products entering the market. Regulators must be equipped with the necessary technology and resources to effectively supervise these emerging distribution channels and ensure that patient safety remains a top priority. —Participant 1, wholesaler*

#### Quality Assurance Challenges

Quality assurance issues emerged as both a potential benefit (see above) and a challenge in the interviews. In terms of challenges, 7 participants identified quality assurance challenges in the current system, such as a lack of authentication technologies. These technologies could include barcode scanners or radio-frequency identification, which allow the tracking of pharmaceutical products throughout the supply chain.

*There are no systems to monitor the products at the end user, the patient, especially in rural areas and among the dense areas … no technologies, no devices. —Participant 1, wholesaler*

#### Technical Challenges

Participants also pointed out that not all innovations are inclusive, meaning they may not be suitable or available to everyone. Some innovative approaches might be out of reach for stakeholders or customer groups that lack the necessary technology. Additionally, the ‘lead time,’ or the time from the start of an innovative project to when it starts showing results, was identified as a challenge. It can take a long time to develop, test, and implement new ways of doing things, which can discourage people from adopting innovative approaches, particularly smaller operations with limited resources.

Participants also noted several underlying technical challenges, including access to basic services, such as electricity and the internet. For example, one participant pointed out that:

*… certain technologies, such as barcodes, can be limited in their usefulness because they require smartphones and electricity to function —Participant 13, innovator*

Finally, participants highlighted that each pharmacy has its own unique set of circumstances, or ‘setup.’ This refers to the specific conditions or context in which a pharmacy operates. For example, a rural pharmacy might not have reliable internet access, which would make certain technologies less useful.

*Sometimes technologies, especially imported tech [technology], aren’t suited to the needs of an organization and its people because each setup is unique. They have to be flexible enough to be adaptable. —Participant 6, regulator*

### Recommendations for Improvement

All participants provided recommendations for improvement to enhance both the current pharmaceutical system and the innovative approaches being implemented in Zambia. Digitization emerged as the most commonly suggested solution, followed by regulatory adaptation and enhanced education/training. Notably, all regulators and professional body representatives emphasized the need for regulatory adaptation.

#### Digitization

Digitization initiatives suggested by participants included improvements such as digitizing patient records and increasing the digitization of the country, to enhance efficiency and effectiveness in the supply chain. Improvements to logistics management were also proposed. Specifically, one recommendation was to introduce an ‘electronic logistics system,’ which would enhance visibility and transparency, making what each facility has issued and dispensed easily analyzable and trackable.

*One recommendation to improve things would be maybe introducing digital patient records and enhancing the country’s digitization efforts are crucial steps towards a more efficient and effective supply chain. —Participant 3, innovator*

#### Education

Education and awareness among stakeholders were also considered necessary improvements. Several participants recommended increasing training for regulators and stakeholders, highlighting the need to train more people to use technologies and have expertise in the processing of waivers and dossiers.

*Increasing training for regulators and stakeholders is essential, as it will enhance their ability to effectively use technologies and manage the processing of waivers and dossiers. —Participant 9, regulator*

#### Government Leadership

Participants underlined the critical role of government leadership in promoting and implementing technological advancements within the pharmaceutical supply chain. A proactive government stance on technology investment is deemed essential for the forward momentum of the innovative pharmacy approach.

*Government leadership is key to the advancement of the innovative pharmacy approach, particularly in the implementation of technology and investment in technological advancements within the supply chain. —Participant 15, supply chain*

#### Regulatory Adaptation

An urgent call for regulatory adaptation reflects the need for a new, accommodative regulatory framework that supports innovative practices in pharmacy. Tailoring regulations to regional peculiarities and establishing stringent measures to ensure medicine quality were highlighted as necessary steps for fostering innovation while safeguarding public health.

*The establishment of a new regulatory framework that is both accommodative and promotes innovative pharmacy practices is crucial. Localized regulations and stringent measures on medicine quality are essential for combating substandard and counterfeit drugs. —Participant 9, regulator*

Stringent regulatory measures were deemed necessary to enforce the quality of medicines, emphasizing that regulations play a pivotal role in curbing the availability of substandard or counterfeit drugs in the market. One suggestion is to provide regulatory allowances for innovative companies to import medicines in bulk.

*This bypasses the limitation of reliance on only a few wholesalers and aligns the supply more closely with the perceived needs of pharmacies. —Participant 7, innovator*

#### Research

Six participants recommended increased research focused on the pharmaceutical supply chain and how innovation impacts the functioning of distribution systems, as well as on the availabality and quality of products for sale.

*There is a pressing need for more research in the field of pharmaceutical supply chains, particularly to understand how innovation can transform systems and improve the quality of medicines. —Participant 13, innovator*

### Overall Concerns

Different patterns emerged across stakeholder groups in their perspectives on the benefits and limitations of innovative private pharmacy distribution channels, as shown in Table 4. These patterns reflect the diverse priorities and challenges faced by each group, shaped by their roles within the pharmaceutical supply chain. For example, innovators consistently emphasized cost barriers as their primary concern, underscoring the financial challenges of implementing new technologies, while regulators focused predominantly on quality assurance, highlighting their responsibility to maintain medicine standards. Wholesalers prioritized distribution efficiency, reflecting their operational focus, and retailers expressed concerns about medicine availability, which directly affects their ability to meet patient needs.

**TABLE 4.** Overall Concerns and Perceptions About Innovative Private Pharmacy Distribution Channels, by Stakeholder Group (N=15), Lusaka, Zambia, 2022

**Stakeholder Group**	**Primary Concerns**	**Secondary Concerns**	**Innovation Stance**
Innovators (n=5)	Cost barriers (5/5)	Regulatory flexibility (4/5)	Highly positive
Wholesalers (n=3)	Distribution efficiency (3/3)	Technology infrastructure (2/3)	Moderately positive
Retailers (n=3)	Medicine availability (3/3)	Implementation costs (2/3)	Positive
Regulator/National agency (n=3)	Quality assurance (3/3)	Authentication systems (2/3)	Cautiously positive
Supply chain expert (n=1)	Technology implementation (1/1)	Infrastructure challenges (1/1)	Positive

## DISCUSSION

This study reveals that innovative private pharmacy distribution channels in Zambia, while offering some potential solutions for medicine access, could most critically impact medicine quality and safety through improved traceability and supply chain transparency. Our findings suggest that the successful implementation of these channels requires a 3-pronged approach: technological integration, government support, and robust regulatory frameworks. Additionally, fostering innovation in medicine registration processes could encourage greater brand diversity, which, in turn, may improve affordability through increased price competition. This integrated approach could transform pharmaceutical distribution with a view to ensuring medicine quality and safety in Zambia’s health care system.

This qualitative study engaged a diverse set of stakeholders, including regulators, pharmaceutical professionals, and health care providers, to understand their perceptions of innovative private pharmacy distribution channels in Zambia. Stakeholders reported both direct and indirect experiences with these channels, acknowledging a range of benefits and challenges. Key advantages, as identified by respondents, include greater accessibility to medications, more efficient distribution and supply chain mechanisms, improved product traceability, and the ability to alleviate various supply chain bottlenecks. However, challenges inherent to many health systems in LMICs, such as the high cost of medicines, frequent stockouts, complex regulatory frameworks, transportation difficulties, and the presence of substandard medications, were also raised. To address these issues, stakeholders suggested the adoption of digitization efforts, comprehensive training programs for regulators and pharmacy staff, proactive government involvement in technology implementation, enhancements in logistics management, regulatory reforms, and further research into pharmaceutical supply chain optimization.

In this study, stakeholder recommendations offer perspectives that are particularly relevant for pharmaceutical supply chains in LMICs. These insights align with existing research that underscores the transformative impact of digitization in streamlining supply chain efficiencies elsewhere. For example, Peltoniemi et al.^24^ have pointed out that digitization has led to more efficient, predictable, and technologically advanced dispensing processes at the pharmacy level in Finland. Digitization is widely acknowledged as a vital mechanism for increasing transparency, traceability, and accountability in the supply chain.^25^^–^^27^ Our study findings add to this discourse by emphasizing the need for customized digital solutions that consider local contexts and unique challenges, a dimension often absent in the current literature.

The potential advantages of innovative private pharmacy distribution channels, particularly for medicine quality and safety, include enhanced product traceability, standardized quality control processes, and real-time monitoring of pharmaceutical integrity throughout the supply chain. While these channels also improve accessibility and affordability, their primary value lies in strengthening quality assurance mechanisms. These findings resonate with existing research that underlines the transformative role of technology in enhancing access to medicine in LMICs. For example, studies by Yadav and Glassman^2^ indicate that technological innovations can significantly improve various aspects of the pharmaceutical sector and the goal of universal health care. Digital innovations, such as mHealth solutions and e-commerce platforms, have the potential to connect pharmacies and medicine manufacturers to consumers, improving accessibility and supply chain efficiency. While specific examples such as last-mile delivery services using drones have been implemented in other LMICs, the innovations observed in Zambia focus more on digitization efforts to enhance transparency and traceability within the pharmaceutical supply chain.^1^^,^^4^^–^^6^

The issue of medicine affordability, highlighted by multiple participants in our study, is consistent with the existing literature that reports elevated drug prices as a persistent challenge in LMICs.^28^^–^^31^ This finding adds further context-specific evidence from Zambia, emphasizing that affordability continues to be a significant barrier to access to medicines. High costs not only limit the availability of medicines in both public and private sectors but also impact their utilization, negatively influencing patient health outcomes. Stockouts in the public sector often force patients to turn to private health care providers, where medicines are typically more expensive, thereby elevating out-of-pocket expenses. This reliance on private health care further exacerbates health care inequalities. Moreover, the financial burden often drives individuals to seek cheaper, yet potentially substandard, alternatives from unlicensed outlets, thereby raising public health concerns.

Our study, along with Miller et al.,^15^ highlights the absence of specific regulatory frameworks for innovative private pharmacy channels in Zambia. This regulatory gap has serious public health implications, as it enables the sale of substandard or falsified medicines, compromising drug quality and patient safety. The urgency to address this issue is underscored by the surge in online medicine purchases during the COVID-19 pandemic.^32^^–^^34^ Lack of regulation jeopardizes the integrity of pharmaceuticals and leads to increased treatment failure, morbidity, and mortality. Technological solutions such as GS1 standards and blockchain offer promising pathways for enhancing medicine quality and mitigating counterfeiting risks.^35^^–^^38^ However, the effective adoption of such technologies depends on robust regulatory frameworks. Lessons can be learned from international regulatory bodies like the U.S. Food and Drug Administration (FDA) and the European Medicines Agency (EMA), and the establishment of the African Medicines Agency^39^ signifies a positive step toward developing a 21st-century regulatory framework to guide Zambia and other member states in ensuring medicine quality. While regulatory challenges were noted as barriers to competition, our findings also highlight a dual potential of innovative approaches. On the one hand, overcoming delays and costs associated with current systems could encourage more manufacturers to enter the market, fostering competition and reducing prices. On the other hand, without adequate regulatory oversight, these innovations could enable dominant firms to consolidate their market positions, creating new forms of monopoly. This balance between fostering competition and avoiding monopolistic outcomes warrants further exploration in policymaking and research.

Our study also emphasizes the critical role of continued education and training for the public, pharmacy staff, and regulators. The technological and regulatory advancements discussed are meaningful only if they are adequately implemented and utilized. This calls for capacity building not only among providers but also among regulators, a point echoed in research by Baber^40^ and Tirivangani et al.^41^ The proactive role of government leadership is imperative for orchestrating these multifaceted improvements in the pharmaceutical supply chain. Only through a harmonized approach, encompassing technological advancements, regulatory reforms, and human capital development, can we hope to tackle the pressing challenges in medicine quality and distribution.

The practical implications of our findings extend beyond theoretical frameworks. Specifically, to support access to quality-assured medicines in Zambia, we recommend: (1) establishing a centralized digital platform for tracking medicine quality throughout the supply chain, (2) developing standard operating procedures for quality control in innovative distribution channels, and (3) creating a regulatory taskforce specifically focused on monitoring medicine quality in these new channels. These practical steps could serve as a model for other LMICs facing similar challenges in pharmaceutical distribution and quality control.

### Strengths of the Study

This qualitative study is the first in Zambia, providing valuable insights into stakeholders’ perceptions and experiences concerning innovative private pharmacy distribution channels and their implications for medicine quality. Our research fills a current knowledge gap and provides an initial framework for further investigation in this area. The rich insights into the complexities, potential advantages, and challenges of these channels highlight the value of qualitative inquiry in less-explored areas, which is crucial for designing effective interventions.

The application of thematic analysis facilitated the systematic identification and elucidation of patterns and themes within the data, enhancing the study’s credibility and ensuring that the findings were grounded in participants’ experiences. The strength of this study lies in the use of individual interviews, which revealed tangible examples and insightful perspectives on the regulation of an innovative pharmacy approach. The diverse range of stakeholders offered a variety of viewpoints, enriching the dataset and leading to a more comprehensive understanding of the topic.

This study sheds light on the potential benefits of innovative pharmacy approaches, including improved product availability, traceability, and procurement efficiency, providing evidence to support their advocacy and implementation. Additionally, the study highlights the complexities associated with the adoption of innovative pharmacy approaches, including behavioral, financial, regulatory, and technical challenges, providing some valuable insights for policymakers and regulators in developing policies and regulations to address these issues.

### Limitations

Although participants were purposively selected to represent diverse stakeholders, their views may not fully represent the wider population involved in innovative private pharmacy distribution channels in Zambia. However, despite the relatively small number of stakeholders interviewed, data saturation was achieved, providing confidence that the findings capture a comprehensive range of perspectives and opinions relevant to this context. Perspectives from groups such as patients and clients were not included, despite their potential importance in understanding end-user experiences and challenges. Future research should incorporate these groups to provide a more comprehensive understanding of these models. Additionally, reliance on self-reported data carries the risk of bias, as participants might have skewed their responses toward what they perceived as socially desirable or been influenced by their unique personal experiences. Potential researcher bias or interpretation is another methodological consideration that may affect the results. Despite the rigorous application of thematic analysis, the researcher’s lens could shape the final outcomes. To address this, the involvement of other authors in reviewing the data and interpretations helped minimize personal bias and ensure a balanced analysis.

### Future Research

Future research should explore key areas to evaluate the impact of innovative private pharmacy distribution channels. Building on the mixed-methods approach undertaken in this PhD research, these areas include longitudinal studies tracking medicine quality indicators before and after the implementation of these channels, comparative analyses of quality control mechanisms between traditional and innovative pharmacy models, and cost-effectiveness analyses of digital traceability systems. Investigating patient and end-user perspectives will provide a deeper understanding of how these models influence medicine quality, access, and affordability. Additionally, periodic studies capturing changes in stakeholder perspectives as these channels evolve will ensure continued relevance of findings. Multi-country studies in other LMICs could help identify scalable best practices for maintaining medicine quality and optimizing pharmaceutical supply chains across diverse contexts.

## CONCLUSION

This qualitative study provides critical insights into how innovative private pharmacy distribution channels could enhance medicine quality and safety in Zambia through improved traceability and supply chain transparency. Despite the challenges of affordability, transportation, and regulatory hurdles, our research highlights the potential for digitization, education, government involvement, and comprehensive regulations to improve the pharmaceutical supply chain, and enhance product availability, quality assurance, and traceability of medicines. These findings have implications for policymakers in Zambia and other LMICs facing similar challenges in pharmaceutical supply chain management. The successful implementation of innovative technologies and practices, if supported by robust regulatory frameworks and government intervention, could transform pharmaceutical distribution systems while ensuring medicine quality and safety. By adopting innovative technologies and practices, countries can work toward improving health equity and access to quality medicines, contributing to global health improvement.

## Notes

**Peer Reviewed**

**First Published Online:** February 24, 2026.
